# Comparative Genomic Analysis Provides Insights into the Phylogeny, Resistome, Virulome, and Host Adaptation in the Genus *Ewingella*

**DOI:** 10.3390/pathogens9050330

**Published:** 2020-04-28

**Authors:** Zhenghui Liu, Hongyan Sheng, Benjamin Azu Okorley, Yu Li, Frederick Leo Sossah

**Affiliations:** 1 Department of Plant Protection, Shenyang Agricultural University, Shenyang 110866, China; liuzhenghui@jlau.edu.cn; 2 Engineering Research Center of Chinese Ministry of Education for Edible and Medicinal Fungi, Jilin Agricultural University, Changchun 130118, China; bazu_okorley@st.ug.edu.gh (B.A.O.); liyu@jlau.edu.cn (Y.L.); 3 Department of Plant Pathology, Washington State University, Pullman, WA 99164-6430, USA; hsheng@wsu.edu; 4 International Cooperation Research Center of China for New Germplasm and Breeding of Edible Mushrooms, Jilin Agricultural University, Changchun 130118, China

**Keywords:** *Ewingella americana*, brown rot disease, needle mushroom, whole-genome sequencing, antibiotic resistance, virulence genes

## Abstract

*Ewingella americana* is a cosmopolitan bacterial pathogen that has been isolated from many hosts. Here, we sequenced a high-quality genome of *E. americana* B6-1 isolated from *Flammulina filiformis*, an important cultivated mushroom, performed a comparative genomic analysis with four other *E. americana* strains from various origins, and tested the susceptibility of B6-1 to antibiotics. The genome size, predicted genes, and GC (guanine-cytosine) content of B6-1 was 4.67 Mb, 4301, and 53.80%, respectively. The origin of the strains did not significantly affect the phylogeny, but mobile genetic elements shaped the evolution of the genus *Ewingella*. The strains encoded a set of common genes for type secretion, virulence effectors, CAZymes, and toxins required for pathogenicity in all hosts. They also had antibiotic resistance, pigments to suppress or evade host defense responses, as well as genes for adaptation to different environmental conditions, including temperature, oxidation, and nutrients. These findings provide a better understanding of the virulence, antibiotic resistance, and host adaptation strategies of *Ewingella*, and they also contribute to the development of effective control strategies.

## 1. Introduction

*Ewingella americana* is a Gram-negative, oxidase-negative, catalase-positive, lactose-fermenting, non-fluorescent, rod-shaped, motile, and facultatively anaerobic bacterium [1]. The bacterium was first isolated from clinical sources and described by Grimont et al. in 1983 [2] as a new genus and species in the family Enterobacteriaceae. The genus *Ewingella* is currently transferred to the class Gammaproteobacteria, order Enterobacterales, and family Yersiniaceae [1]. *E. americana* is the only known species in the genus. The bacterium has a wide range of hosts, including human [3], mollusks [4], plants [5,6], vacuum-packed meat [7], nutria carcasses [8], and mushrooms [9,10,11]. In mushrooms, *E. americana* is known to cause internal stipe necrosis in *Agaricus bisporus* [9,12] and brown rot on cultivated *Flammulina filiformis* [10].

*E. americana* has emerged as a global problem with reports from many regions of the world, including the United States [13], Spain [14], Kingdom of Saudi Arabia [15], Egypt [16], and China [6,10]. It is generally resistant to many antibiotics. To date, six *Ewingella* species’ genome sequences have become publicly available at the National Centre for Biotechnology Information (NCBI) genome database. However, few studies have been done on the comprehensive analysis of *E. americana* genomes. Consequently, the pathogenicity, functional roles, metabolic capabilities, and the genetic adaptation to its hosts remain unknown.

Next-generation sequencing technologies have expedited sequencing and increased the number of bacterial genomes [17]. The advent of bioinformatics tools in recent years has aided comparative analysis within different taxon levels of microorganisms [18]. Genomic comparisons can address issues in taxonomy, phylogenetics, virulence, and genotype resistance profiles from different hosts [19,20,21]. It can also facilitate a better understanding of the specific mechanisms deployed by the bacteria to adapt to different hosts and environmental conditions. 

In this study, we used Single-Molecule-Real-Time (SMRT) technology [22] to generate the whole genome sequence of *E. americana* B6-1 isolated from cultivated *F. filiformis*. The genome sequence was annotated and compared with the representative genomes of other *E. americana* strains isolated from different hosts/habitat to assess their phylogeny, pan- and core-genome, virulence, antibiotic resistance genes, mobile genetic elements, and defense system. The comparison of the five *E. americana* genomes will enhance our knowledge of pathogenicity and adaptability to different hosts. 

## 2. Results

### 2.1. Genomic Features of E. americana B6-1

**Figure 1:**
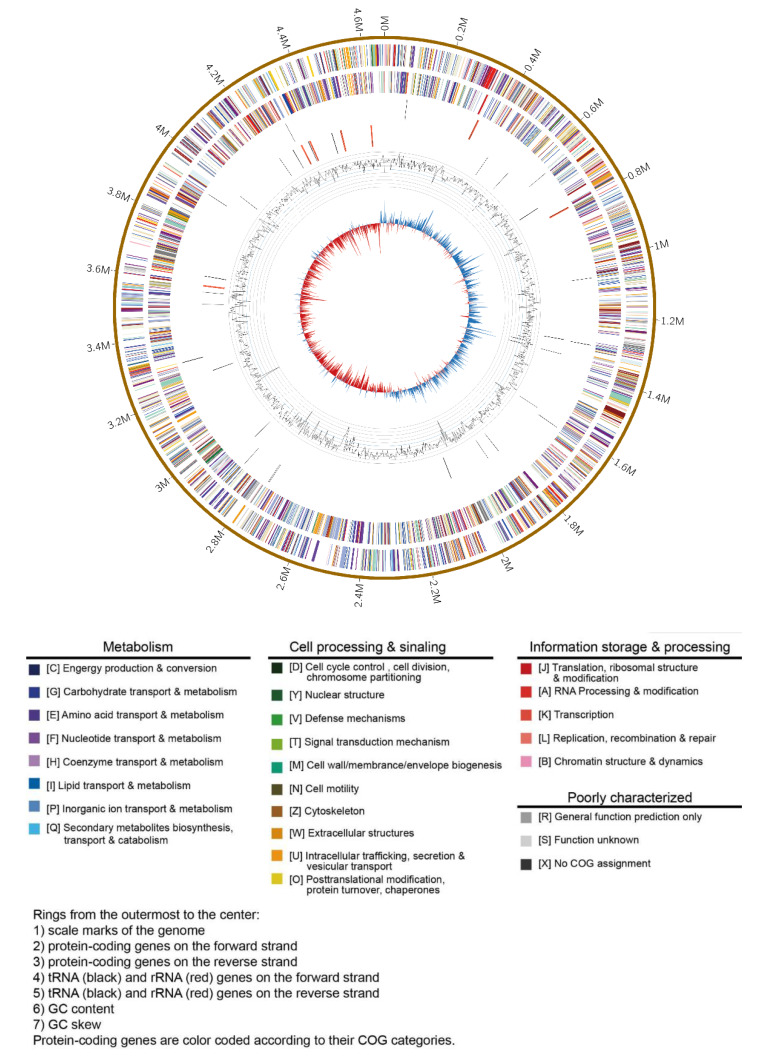
Circos plot of *E. americana* strain B6-1 genome showing the densities of GC (guanine-cytosine) content, tRNA, rRNA, and protein-coding genes.

Strain B6-1 was confirmed as *E. americana* from the phenotypic, biochemical characters (Supplementary Table S1), and the 16S rRNA gene phylogenetic analysis from previous work [10]. The whole-genome sequence of *E. americana* B6-1 was assembled into one circular chromosome (4.67 Mbp in size with GC content of 53.80%) and two plasmids (330 Kbp and 104 Kbp in size with GC content of 53.26% and 50.98%, respectively) (Figure 1). The N50 and L50 were 18,509 bp and 13,069 bp, respectively. Total 4301 protein-coding sequences (CDS) and 99 RNA genes (including 77 tRNA, eight 5S rRNA, seven 16S rRNA, and seven 23S rRNA genes) were detected from the assembled genome. The protein-coding genes were assigned to 25 clusters of orthologous groups (COG) and functional categories. 

### 2.2. Average Nucleotide Identity Calculations and Phylogenetic Analyses

**Figure 2:**
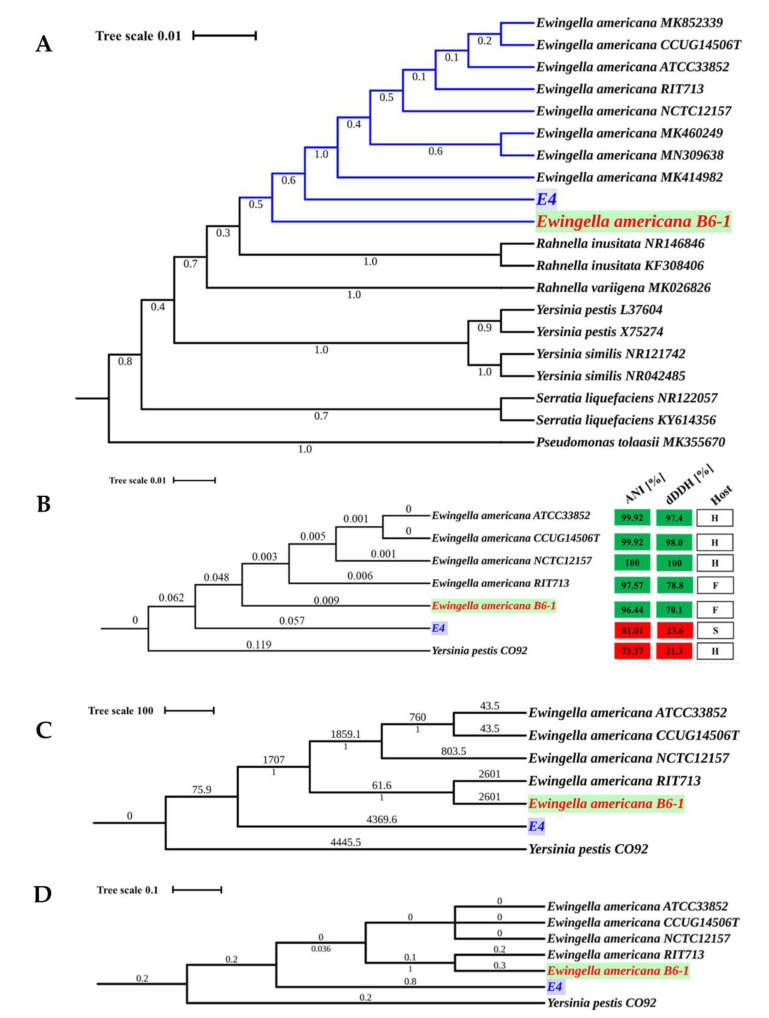
Phylogenetic analyses of the five strains of *E. americana* and other related species. (**A**). Maximum-likelihood phylogenetic tree computed using 16S rRNA gene sequences of the five *E. americana* strains studied. (**B**). Maximum-likelihood phylogenetic tree using 2,095 core- proteins for the five strains of *E. americana* and other related species generated with 1000 bootstrap replications. (**C**). Whole-genome MLST (wgMLST) using 10,373 alleles for the five strains of *E. americana* and other related species (**D**). Phylogenetic tree based on the SNPs of the five *E. americana* strains and other related species. *Yersinia pestis* CO92 was used as an outgroup and root for trees (**B**–**D**). *Pseudomonas tolaasii* was used as an outgroup and root for trees (**A**).

The 16S rRNA-based phylogeny and whole genome-based phylogeny were produced to determine the phylogenetic relationship among the *E. americana* strains. The strains had similar 16S rRNA gene sequences with 98.97 to 100% similarity. The maximum-likelihood (ML) phylogenetic tree using 16S rRNA gene sequences placed all the five strains in the genus *Ewingella* (Figure 2A).

The whole genome-based phylogeny consisted of a core set of 2095 single-copy orthologues proteins for the genus (Figure 2B), whole-genome multi-locus sequence typing (wgMLST) (Figure 2C), single-nucleotide polymorphism (SNP) (Figure 2D), and family trees (Supplementary Figure S3), respectively. The genus core-proteins, wgMLST, and SNP trees showed that the *E. americana* strains (ATCC, CCUG, and NCTC) isolated from humans are more closely related than the other two strains. The wgMLST and SNP trees clustered the strains according to their host, but the genus core-protein tree clustered the strains according to their evolution. All the whole-genome based trees showed that strain E4 is the ancestor of *E. americana* strains. 

Moreover, the genetic relatedness among the five *E. americana* strains in the phylogenetic analyses was confirmed by results from the average nucleotide identity (ANI), and digital DNA-DNA hybridization (dDDH) values based on the genomic sequences. The pairwise ANI and dDDH values ranged from 81.01% to 100%, and 23.6 to 100%, respectively. The detailed results of ANI and dDDH values are presented in Supplementary Table S4. The ANI and dDDH values of strain E4 (81.01% and 23.6%) were well below the defined thresholds for species delineation, 95–96% for ANI, and 70% for GGDC. The Tetra Correlation Search (TCS) calculations for strain E4 was related to *Serratia* sp. Leaf51 (Supplementary Table S5). However, the 16S rRNA sequence showed higher similarity to *E. americana* compared to *Serratia* sp. Leaf51. Besides, there was slightly more variability in the ANI and dDDH values between E4 and the *E. americana* (81.01% and 23.6%) compared to *Serratia* sp. Leaf51 (78.92% and 22.2%). Since strain E4 is probably a new species, it was not used in the subsequent analyses. 

### 2.3. Orthology and Pan-Genome Analysis

**Figure 3:**
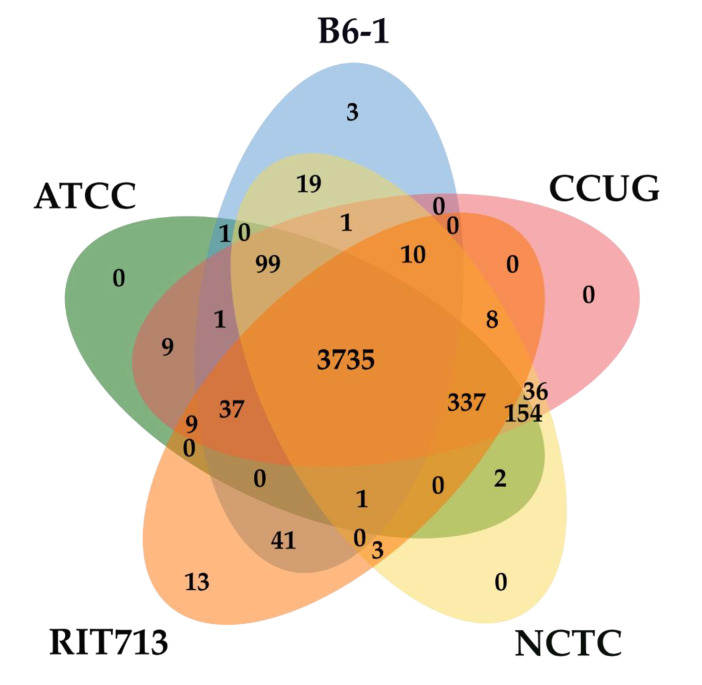
Venn diagram of the unique and shared number of orthologous gene clusters among the genomes of the five *E. americana* strains.

The orthologous gene clusters shared among the five strains of *E. americana* were identified. All five strains of *E. americana* formed a total of 4519 clusters and shared 3735 orthologous clusters (Figure 3). The singletons ranged from 5 to 790 gene clusters. The unique orthologue gene clusters within the species were 13, 3, and 0 for RIT713, B6-1, and other three strains, respectively. Strain B6-1 shared a higher number of orthologous clusters with RIT713 (41) compared to the other strains 

Functional analysis of the gene clusters showed that biological processes were the most assigned Gene Ontology (GO) terms. A total of 10,359 shared orthologous gene clusters were assigned to biological processes GO terms within the five *E. americana* strains. Some of the GO annotations among the core shared orthologous proteins include glycogen catabolic process (GO:0005980), anaerobic respiration (GO:0009061), pathogenesis (GO:0009405), rhamnose catabolic, and metabolic processes (GO:0019301 and GO:0019299) and terpenoid biosynthetic process (GO:0016114). Oxidoreductase activity (GO:0016491; *p*-value = 9.47 × 10^−5^) was the only enriched GO term for the shared orthologous gene clusters among the five strains.

The GO enrichment analysis between B6-1 and RIT713 revealed enriched GO terms for the lipopolysaccharide core region biosynthetic process (GO:0009244; *p*-value = 4.48 × 10^−5^) and cellular response to DNA damage stimulus (GO:0006974; *p*-value = 4.48 × 10^−5^). Protein secretion by the type II secretion system (*p*-value = 1.85 × 10^−12^) was the enriched GO term found in the unique gene cluster of RIT713. The annotation of unique orthologous clusters in B6-1 showed classification in biological processes as the main category, with genes related to the biological process, metabolic process, toxin metabolic process, secondary metabolic process, cellular metabolic process, and heterocycle metabolic process as a subcategory. The toxin metabolic process contains two protein-coding genes involved in the aflatoxin biosynthetic process (GO:0045122).

The *E. americana* pan-genome (Supplementary Table S6) for the five strains contained 5103 gene families, and 43 to 421 new genes were found in four genomes (B6-1, CCUG, NCTC, and RIT713). The genes of the pan-genome increased from 4275 to 5104, and core genes decreased from 4275 to 3677 with the addition of a new genome sequence. The pan-genome curve (Supplementary Figure S2) did not reach the plateau by the addition of new genes with each additional genome. The expansion parameter ’b’ was 0.102 (>0).

### 2.4. Resistome and Antimicrobial Susceptibility Profile of B6-1

A total of 27 unique antibiotic resistance genes were identified in all the genomes of the *E. americana* strains, with four different mechanisms of resistance from the comprehensive antibiotic resistance (CARD) database. The multiple antibiotic resistance genes (ranging from 20 to 33) in the strains (Supplementary Table S7) were associated with an aminoglycoside, aminocoumarin, carbapenem, cephalosporin, diaminopyrimidine, fluoroquinolone, fosfomycin, macrolide, nitroimidazole, a peptide antibiotic, phenicol, rifamycin, and tetracycline antibiotic. Fosfomycin resistant gene (*fosA*) was found in all the strains. The most abundant antimicrobial resistance gene families were encoding multi-efflux pump (23 genes).

Strain B6-1 was tested for antibiotic susceptibility (Table 1) by the Kirby–Bauer test. It showed resistance to ampicillin, cefazolin, clindamycin, novobiocin, rifampicin, tetracycline, and vancomycin. However, B6-1 showed intermediate resistance to cefixime and erythromycin, but it was susceptible to aztreonam, ceftriaxone, ciprofloxacin, gentamicin, kanamycin, ofloxacin, and streptomycin.

### 2.5. Mobile Genetic Elements (MGE) 

From the INTEGRALL database search, four and five putative class 1 integron (*In1*) genes with a variety of cassette arrays (Table 2 and Supplementary Table S9) were found in the genomes of (B6-1, ATCC, CCUG, and NCTC) and RIT713, respectively. All the five *E. americana* strains contained one class 1 Integron (In1) with *catB8j-aacA4-aadA5* cassette arrays. Class 1 integron (*In1*) with cassette arrays *dfrA12-gcuF-aadA2* and *dfrA14b-arr-2-cmlA5-blaOXA-10-aadA1-qacED1-sul1* were only found in B6-1 and RIT713. 

A total of four different insertion sequence (IS) families were detected in all the five genomes of *E. americana* strains (Supplementary Table S10). IS*3* and Tn*3* were the dominant IS families. All the *E. americana* strains (ATCC, CCUG, and NCTC) isolated from humans contained four IS gene families, while those isolated from mushrooms (B6-1 and RIT713) had the least number of IS families (n = 2). Among the *E. americana* strains, only B6-1 possessed plasmids (n = 2). The plasmids *pB61a* and *pB61b* were 330 kb and 104 kb in size, respectively. Both plasmids contained genes coding for antibiotic resistance, insertion sequences, and toxin-antitoxin system (Table 2 and Supplementary Table S11). 

Intact phages were found in all the strains (Table 3). All the phages were circular excerpt ATCC33852, which had linear phage. Strain B6-1 contained two sequences with two phage regions. From the IslandViewer4, the five genomes of *E. americana* strains contained 19 to 24 genome islands (GIs) (Table 2) of total length ranging from 8 to 10 kb. The gene annotation showed that most of the genes were hypothetical proteins with unknown function, while other genes were associated with replication, recombination, repair, integrases, transposases, and other genome mobility-related genes. 

### 2.6. CRISPR-CAS System, Restriction Modification System, and Toxin-Antitoxin System

A total of five (ranging from 2 to 5 spacers) clustered regularly interspaced short palindromic repeats (CRISPR) encoding type I CRISPR-Cas systems were identified in the genomes of the *E. americana* strains through CRISPRCasFinder (Table 2). ATCC33852 had the highest number of CRISPR spacers (n = 5). Strains B6-1, CCUG, and NCTC had four CRISPR spacers. None of the strains contained the Cas element. 

All the strains contained putative genes for the type II restriction-modification system (R-M system). Type I R-M system was found in four strains (ATCC, B6-1, CCUG, and NCTC), and the type IV R-M system was found in only two strains (B6-1 and RIT713). Putative genes for the toxin-antitoxin system (TA) were found in all the *E. americana* strains (Supplementary Table S12). Complete type II and type IV TA gene modules were found in all the genomes of the five *E. americana* strains. Type I TA gene modules were present in only strains B6-1 and RIT713. 

### 2.7. Pathogenicity and Virulence Factors

The genomes of the five *E. americana* strains were surveyed to investigate pathogenicity and virulence-associated genes. Strain B6-1 had a predicted probability score (P score) of 46.24 and the probability of being a human pathogen of 0.60. It was matched to 24 pathogenic families. The predicted probability score (P score), probability of being a human pathogen, and pathogenic families matched to all the *E. americana* strains ranged from 46.21–53.60, 0.60–0.63, and 24–25, respectively (Table 4). The five *E. americana* strains (B6-1, RIT713, ATCC, CCUG, and NCTC) were all predicted as a human pathogen. 

From the virulence factor database search, a total of 82 putative genes virulence-associated were found in genomes of the five *E. americana* strains (Supplementary Table S13). Out of the 82 genes, those found in each genome ranged from 66 to 70 genes. B6-1 contained 67 putative virulence genes. The most abundant virulence features in the genomes of all the five *E. americana* strains were secretion system, adherence, invasion, chemotaxis and motility, and immune evasion. Pore-forming toxins were found in all the five genomes of the *E. americana* strains. 

Further examination of the macromolecular secretion system revealed the *E. americana* strains encode putative genes related to flagella, Type I, Type III, Type IV, Type V and Type VI secretion systems (Supplementary Table S14). The total number of putative genes for the macromolecular secretion system among the strains ranged from 136 to 199. The least and most abundant macromolecular secretion putative genes were found in B6-1 (136 genes) and RIT713 (199 genes), respectively. The most abundant macromolecular secretion system was type VI, followed by Type I, Type II, and Type III secretion systems. Type II secretion genes (*gspD*, *gspE*, *gspF*, *gspG*, *gspM*, *gspH*, *gspI*, *gspJ*, *gspK*, and *gspC*) were found in RIT713.

### 2.8. Stress Response 

Genes associated with stress response were identified in all the five strains of *E. americana*. Strain B6-1 had the highest number of putative genes (90) for stress response compared to the other strains (86–87 genes). Genes coding for oxidative stress (35.40%) followed by stress response (21.60%) and osmotic stress (17.06%) were the most abundant in the genomes of the five strains of *E. americana*. A persister cell-related gene (cell division inhibitor *SulA*) and sporulation associated gene (peptidyl-tRNA hydrolase (EC 3.1.1.29)) were found in all five strains (Supplementary Table S15). In addition, five genes coding for sulfate and thiosulfate import ATP-binding protein CysA (EC 3.6.3.25), DedA protein, S-formylglutathione hydrolase (EC 3.1.2.12), TsgA protein and S-(hydroxymethyl) glutathione dehydrogenase (EC 1.1.1.284) for detoxification, and a cold shock protein *CspA* for cold stress tolerance were also found in all the genomes of the five *E. americana* strains. 

### 2.9. Annotation of Carbohydrate-Active Enzymes (CAZymes)

**Figure 4:**
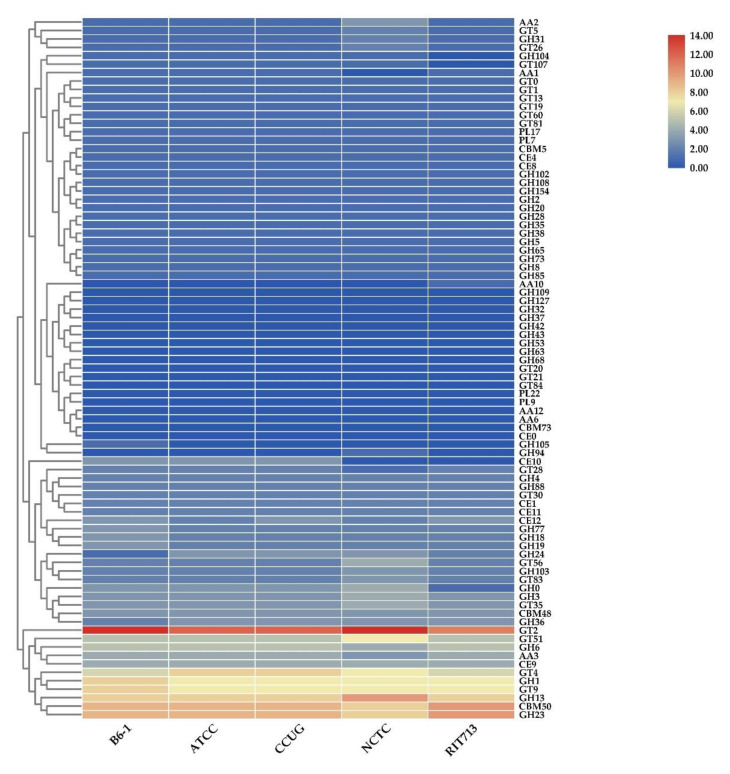
Heatmap showing the abundance of each CAZyme family in each genome of the *E. americana* strains.

The presence of carbohydrate-active enzymes (CAZymes), including the variety of families of glycoside hydrolases (GHs), glycosyltransferases (GTs), polysaccharide lyases (PLs), carbohydrate-binding molecule (CBM), carbohydrate Esterase (CE), and auxiliary activities (AA), that synthesis, metabolize, and transport carbohydrates were identified using the dbCAN2 meta server. Table 5 shows the total number of predicted CAZymes genes that were found in the genomes of the five *E. americana* strains. The total number of CAZymes ranged from 151 to 168. Strain B6-1 contained 160 CAZymes, while the most abundant and least CAZymes were found in NCTC and RIT713, respectively. The large average sets of genes of CAZyme families among the pathogens were GH 43.43% (68.8 genes) and GT 33.46% (53 genes). GT2 (chitin synthase (EC 2.4.1.16)) was the most abundant CAZyme module (Figure 4). Other abundant GT modules are GT4 (sucrose synthase (EC 2.4.1.13)), GT9 (lipopolysaccharide N-acetylglucosaminyltransferase (EC 2.4.1.56)) and GT51 (murein polymerase (EC 2.4.1.129)). The abundant GH modules among the genomes of the five *E. americana* strains were GH23, GH13, and GH1. Two to three genes encoding GH18 (chitinase (EC 3.2.1.14)) were identified in the genomes of *E. americana* strains. GH75 (chitosanases) were absent in all the genomes of *E. americana*. The genomes of *E. americana* strains contained a large number of CBM 50 (8-10 genes), which are found to be attached to various enzymes from families GH18, GH19, GH23, GH24, GH25, and GH73, to degrade the chitinous fungal or peptidoglycan bacterial cell walls. All the genomes encode PL17, a putative alginate lyase (EC 4.2.2.3), or oligoalginate lyase (EC 4.2.2.26) and PL7 (poly(β-mannuronate) lyase/M-specific alginate lyase (EC 4.2.2.3)). The *E. americana* genomes encoded putative genes for CE1, CE4, CE8, CE9, CE11, and CE12, which degraded xylans, chitin, peptidoglycan, pectin, N-acetylglucosamine (GlcNAc), and lipids. 

### 2.10. Secondary Metabolites and Bacteriocins

The genomes of the five *E. americana* strains were searched against the antibiotics and secondary metabolite analysis shell (antiSMASH) 5.0 (Supplementary Table S16) and BAGEL 4 web server. Five and seven putative biosynthetic gene clusters were found in four (ATCC, CCUG, NCTC, and RIT713) and B6-1, respectively. Three known secondary metabolites, aryl polyenes, O-antigen, and desferrioxamine E, which are arylpolyene, thiopeptide, and siderophore, respectively, were commonly found among all the five *E. americana* strains. The other two to four gene clusters have no known annotation. The *E. americana* genomes contained multiple genes related to bacteriocin production (bottromycin, colicin-M, and microcin). Bottromycin was found in the five genomes. Microcin was found in four genomes (ATCC, CCUG, NCTC, and RIT713) and plasmid *pB61a* of strain B6-1. Colicin-M was found in only two genomes (B6-1 and RIT713). 

## 3. Discussion

*Ewingella americana* is known to cause disease in hosts from different kingdoms, and there is an emergence of its multi-drug resistance worldwide [13,15,23]. The study aimed to examine the phylogeny, resistome, mobilome, virulome, and defense systems of five strains of *E. americana*. The genome size and the number of genes of strain B6-1 were slightly small but comparable to those in the genus. The differences in the genome sizes and the number of genes may be due to the influence of mobile genetic elements [24].

The 16S rRNA gene-based phylogenetic tree showed that all the strains were related to *E. americana*. However, E4 showed ANI and dDDH values were below the acceptable threshold used to differentiate the closely related species [25]. The genome-based phylogenetic trees for the *E. americana* corroborates the ANI and dDDH values. Therefore, strain E4 should be reclassified as a different species based on morphological and physiological characteristics. The results confirm that 16S rRNA gene sequences alone cannot resolve the phylogeny of bacteria at the species or genera level [26]. The results suggest that morphological, biochemical, 16S rRNA gene sequence, as well as genome-based comparative, remain essential in delineating bacterial taxa [27]. 

The highly conserved orthologous genes mean all the strains evolved through speciation events from the last common ancestor [28]. Annotation of the conserved orthologous genes revealed they play an active role in essential biological processes, metabolic functions, and cellular processes. The differences in accessory and unique genes among the *E. americana* strains confirms the previous report that strains within a bacterial species usually have a set of conserved core genes and a variable set of accessory genes [29]. 

Interestingly, annotation of the unique clusters in B6-1 revealed putative genes related to the biosynthesis of toxins, namely aflatoxin. Aflatoxins are carcinogenic and mutagenic secondary metabolites produced by members of *Aspergillus* sp. (commonly by *Aspergillus flavus* and *A. parasiticus*) that contaminate many food crops [30]. Besides fungi, bacteria residing within the fungal cytosol could produce mycotoxins, such as rhizoxin and rhizonin [31]. We speculate that B6-1 could have acquired the putative aflatoxin genes from mushroom substrate contaminated with *A. flavus* and *A. parasiticus* [32] for self-defense against other predatory fungi. Therefore, strain B6-1 has the potential to contaminate mushrooms by producing aflatoxins. This result suggests the need for effective control of *E. americana* and other pathogenic fungi in mushroom production and food processing to prevent the adverse effect on human health.

The *E. americana* strains showed an open but soon to be closed pan-genome. The results indicate that every newly sequenced genome contributed new genes to the species, but the availability of a large number of complete genomes for this species will fail to add new genes to the *E. americana* pan-genome. Genome size, protein-coding genes, lifestyle, isolated niche, and natural environment of bacteria may influence the size of the pan-genome [33]. The *E. americana* strains from this study were isolated from different hosts, habitat, and environment; hence, they might have influenced the pan-genome. The pan-genome analysis suggests that additional high-quality reference genomes representing different eco-species may provide a better understanding of the biology of *E. americana*. 

The putative antibiotic resistance genes found among all the strains confirm that *E. americana* were frequently resistant to several classes of antimicrobial agents [3,15,23]. The predominance of antibiotic resistance genes in the core and accessory genomes of the strains in this study may be associated with the evolution of multidrug resistance in the taxa [34,35,36] and the ability to successfully acquire antibiotic resistance genes encoded by mobile genetic elements such as insertion sequences, transposons, integrons, and plasmids from their host or different bacterial cells [37]. The *E. americana* strains may be out-competing strains with lower resistance multiplicity in their habitat [34].

In addition, increased use of antibiotics in healthcare and animal farms, as well as in the mushroom industry, could have led to the emergence, spread, and persistence of multidrug-resistant *E. americana* strains [36]. From the orthology analysis, rhamnose metabolism was found in all the *E. americana*. Therefore, new therapeutic compounds targeting rhamnose biosynthesis can be used to control the pathogen [38]. 

Virulence genes play an important role in pathogenicity, and several of such genes were found in the genomes of all the *E. americana* strains in this study. In silico prediction of the pathogenic potential revealed that about 60% of the pathogenic families were linked to the family Yersiniaceae. The pathogenicity of the *E. americana* strains (B6-1, ATCC, CCUG, NCTC, and RIT713) are weak pathogens of humans compared to the type species in the family Yersiniaceae, *Yersinia pestis* CO92 (1569 matched pathogenic families). This result confirms which *E. americana* is an opportunistic pathogen that infects immunosuppressed patients due to other illnesses [13,39]. However, the predictive method is not sufficient to arrive at a conclusion about the pathogenesis of the microorganism [40]. Therefore, there is a need to conduct further pathogenicity testing to confirm the pathogenesis of all the other four *E. americana* strains. The virulence factors found in the core genome, such as flagella, pili, and type secretions, were mostly conserved across the strains and were involved in adherence and immune system evasion. The virulence genes are ubiquitous as they likely play a role in the fitness of *E. americana* in different environments, whereas accessory virulence factors offer additional functions for improved environmental fitness. The acquisition of plasmids by B6-1 could increase its virulence. The *E. americana* strains’ putative toxins may play a significant role in its pathogenesis and survival. 

The identification of only class 1 Integron and the abundance of IS3 and Tn3 family elements in *E. americana* strains confirms the report that they are the most widely distributed mobile DNA elements in bacteria and contribute to the dissemination of antibiotic resistance and the emergence of multi-resistant pathogens worldwide [41,42,43]. In addition, the acquisition of two plasmids by strain B6-1, absent in the other strains, may play an essential role in virulence, antibiotic resistance, detoxification, and ecological interaction [44]. The results suggest that the various MGEs influenced the size of the genome islands among the strains, hence the varied number of virulence or resistance genes between the strains.

Bacteria have developed multiple systems, including CRISPR–Cas systems and restriction-modification (R-M) systems, to defend themselves against invaders such as plasmids or phages [45]. This result corroborates that multiple CRISPR elements can often be detected in bacterial genomes, but not all elements are accompanied by Cas genes [46]. However, the intact phages in the genomes of the other five *E. americana* strains could be due to the development of anti-CRISPR systems by phages to avoid CRISPR regulation to enable integration into the genome [47]. It is not surprising to find genes related to R-M systems (type I, II, and IV) among the *E. americana* strains because R-M systems are widespread and considered as an effective immune system in bacteria and archaea [48,49]. The two to three R-M systems among the strains are consistent with other bacteria. The numerous and diverse R-M systems in the *E. americana* strains isolated from mushrooms (B6-1 and RIT713) may provide a selective advantage by rapid genetic adaptation to its natural environment [50].

Further, the stress related genes found in the genomes of *E. americana* were targeted for a particular kind of stress. The results suggest all the *E. americana* strains can adapt to any stress, including tolerance for oxidative, osmotic, carbon starvation, nutrients, cold, and heat stress. Also, they possessed toxin-antitoxin modules, which play significant roles in persister formation when exposed to environmental stimuli [51]. TA systems are gene modules that encode a protein toxin and an antitoxin that neutralizes either the toxin’s action or its expression [52]. The abundant and complete type II TA system found among the strains confirms the reports of its wide distribution and diversity [53]. TA system is primarily involved in biological processes such as DNA replication, mRNA synthesis, cell wall synthesis, and programmed cell death [54]. Therefore, it could serve as a potential target for novel antimicrobial agents [55] to control multi-drug resistant bacteria like *E. americana*.

The diverse repertoire of putative CAZyme genes found in all the *E. americana* strains indicates that complex enzymes are produced to digest the components of their host’s cell wall, including complex polysaccharides (cellulose, hemicellulose, and pectin) in plants [56] and chitin in fungi, insects, and mollusks. The difference in CAZyme numbers and the presence or absence of some CAZyme families may indicate different substrate utilization capabilities. There were chitinase GH18 in all the strains, indicating that they share similar strategies for degrading fungal cell walls. However, strain B6-1 had more of GH18 and GH19. This suggests that it may require more chitinase to invade its fungal host. All the *E. americana* strains produced abundant GT2 and CMB50, which are important for evading animal/plant and fungi/plant cells, respectively [57,58]. CMB50 acts as a chitin surface binder for the plant or fungal hosts’ invasion and colonization [59]. The secreted CAZymes in the genomes of *E. americana* strains are potential virulence factors, particularly for the host fungi and plants. Actual experiments are required to validate and confirm the designation [59].

All the *E. americana* strains possessed genes for biosynthesis of secondary metabolites, which are not essential for growth, development, or reproduction of the organism but have an important ecological function [60]. The large number of secondary metabolite gene clusters in strain B6-1 may be required for its survival in the mushroom environment. The O-antigen cluster found in the strains may be required for virulence [61] and resistance to complement-mediated killing and phagocytosis [62]. The siderophores, desferrioxamine E (found in all the strains) may play an essential role in bacterial pathogenesis by scavenging iron from the host or their surrounding environment [63,64]. The siderophores may also be involved in oxidative stress tolerance and have applications in medicine and agriculture [63]. All the *E. americana* strains produced aryl polyene pigments, and they were similar to carotenoids [65,66]. Some bacterial species, such as *Enterococcus mundtii*, are known to produce carotenoid-like pigments [40]. Aryl polyene pigments play a role in protecting the bacteria from oxidative stress when exposed to the environment [66,67]. All the strains also produced bacteriocin. The production of colicin M toxin may be relevant and unique characteristics for strains B6-1 and RIT713, which were isolated from fungi. The identified putative gene-encoded antimicrobial peptides (bottromycin, microcin, and sactipeptide) and colicin M toxin may be responsible for microbial competition [67,68,69]. The genome mining of secondary metabolites from the *E. americana* suggests the potential to use the bacteriocins in healthcare, animal husbandry, and the food industry as well as agriculture, to replace antibiotics and to treat multi-drug resistant pathogens [70,71]. However, further work needs to be done using other detection techniques like chromatography to ascertain the production of the secondary metabolites, bacteriocins, and assessment of their efficacy for controlling multidrug-resistant pathogens.

## 4. Materials and Methods 

### 4.1. Bacterial Strains and Characterization

*Ewingella americana* strain B6-1 was isolated from a symptomatic mushroom, *Flammulina filiformis*, collected from the Gaorong Biotech Company at Changchun, Jilin, China, in 2016. The other four *E. americana* strains used in this study include three strains isolated from the human throat (*E. americana* ATCC 33852 [72], *E. americana* CCUG 14506T [73], and *E. americana* NCTC12157 [74]), and one from mushroom (*Craterellus* sp.) *E. americana* RIT713 [75]. In addition, strain E4 [76], isolated from permafrost soil, was included to ascertain its name and taxonomic position. There is no report of the pathogenicity test for the five other strains.

The isolation and identification of *E. americana* B6-1 were described in the previous report [10]. Molecular characterization was done by amplifying 16S rRNA and *gyrB* genes with the primers 27F/1492R and gyrB-UP1s/gyrB-UP2sr, respectively, using polymerase chain reaction (PCR). The colony morphology of B6-1 was observed after 72 h growing at 28 °C under the dark on nutrient agar (NA), blood agar, and Kings Medium B (KB), separately. Bacterial cell morphology was examined using a transmission electron microscope (HITACHI H-7650) after 48 h growing on NA media.

For biochemical and physiological tests, API 20E, and API 50CHE kits (BioMérieux, Marcy-l’Etoile, France) were used following the methods described by Mergaert et al. [77]. The bacterium was grown at 30 °C overnight in 5 mL Luria-Bertani (LB) liquid medium. The bacterial cells were centrifuged, collected, and washed twice with sterile distilled water. The API assays were repeated three times. The pellets of bacterial cells were tested by Qingdao Kechuang Quality Testing Co. LTD. (Qingdao, Shandong Province, China) for Fatty acid methyl esters (FAME) analysis with an Agilent Technologies 7890A Gas Chromatograph using methods described by Ivanovic et al. [78].

### 4.2. DNA Extraction, Genome Sequencing, and Annotation

The genomic DNA was extracted from *E. americana* B6-1 grown overnight in 20 mL LB broth at 28 °C using BioFlux Biospin bacterial genomic DNA extraction kit (Bioer Technology Co., Ltd., Hangzhou, China). The DNA quality was examined by gel electrophoresis and quantified using the Qubit 2.0 fluorometer (Life Technologies, Darmstadt, Germany). The DNA was used to construct a Single-Molecule Real-Time (SMRT) Bell library with an insert size of 20 kb at Tianjin Biochip Corporation (Tianjin, China). The library was sequenced using the PacBio RSII platform (Pacific Biosciences, Menlo Park, CA, USA). The low-quality reads were filtered out by the SMRT analysis software 2.3.0 [79], and the filtered reads were de novo assembled by a hierarchical genome assembly process (HGAP) [79] with the SMRT portal software. 

The genome annotation was done by Prokka, prokaryotic genome annotation software [80]. Functional annotation of the genes was performed by the BLASTP search against the Cluster of Orthologous Groups of proteins database (COG, https://www.ncbi.nlm.nih.gov/COG/) [81], and the Kyoto Encyclopedia of Genes and Genomes database (KEGG, http://www.genome.jp/kegg/) [82]. Gene ontology (GO) was analyzed using InterProScan 5 [83,84]. The genome of *E. americana* B6-1 was deposited in the GenBank database under the accession CP048243 in the Genome and SAMN13930952 in the BioSample. In addition, four other *E. americana* genomes (including *E. americana* ATCC 33852 [72], *E. americana* CCUG 14506T [73], *E. americana* NCTC12157 [74], and *E. americana* RIT713 [75]), and a wrongly named species E4 [76] (Supplementary Table S3) were downloaded from the National Centre for Biotechnology Information (NCBI) (https://www.ncbi.nlm.nih.gov/) genome repository and reannotated following the same pipeline as B6-1. The quality of all the genome assembly was assessed using Quast (v5.0.2) [85].

### 4.3. Average Nucleotide Identity and Phylogenetic Analyses

The Average Nucleotide Identity (ANI) was calculated using ANI Calculator (https://www.ezbiocloud.net/tools/ani) [86], and the digital DNA-DNA hybridization (dDDH) was calculated using Genome-to-Genome Distance Calculator 2.0 (GGDC) server (http://ggdc.dsmz.de/ggdc.php#) [87]. Additionally, Tetra Correlation Search (TCS) was used to search strain E4 genome against the entire genomes reference database (GenomesDB) of JSpecies Web Server (http://jspecies.ribohost.com/jspeciesws/) [88] to provide insights into the relationships to other organisms. 

The phylogenetic relationship among the *E. americana* strains and its closest neighbors was determined by the whole genome-based and 16S rRNA sequences (Supplementary Table S2). The 16S rRNA sequences of each *E. americana* strain were obtained from the NCBI GenBank database and aligned using ClustalW application in MEGA X [89]. A phylogenetic tree of the 16S rRNA gene was constructed using the maximum likelihood method based on the JTT matrix-based model [90] with 1000 bootstraps replications in MEGA X [89]. OrthoMCL 2.0.9 [91] was used to cluster the protein sequences of each *E. americana* strain. Each set of orthologous proteins were individually aligned using MUSCLE [92]. The poorly aligned and divergent positions of protein sequences were trimmed with Gblocks v0.19b [93]. The final conserved blocks were concatenated to create a core-proteome alignment and used for the construction of a maximum likelihood phylogenetic tree by the LG matrix, and the Gamma model of rate heterogeneity [94] with bootstrap supporting of 1000 replicates in RAxML v8.0.29 [95]. 

The whole-genome multi-locus sequence typing (wgMLST) tree was constructed using the PGAdb-builder web service [96]. The PGAdb profile of five *E. americana* strains and two other related species genomes was compared by BLASTn, with 90% coverage and 90% identity. The SNP-based phylogenomic tree was constructed using the CSI Phylogeny-1.4 (https://cge.cbs.dtu.dk/services/CSIPhylogeny/) web service [97]. *Yersinia pestis* CO9 genome sequence was used as an outgroup for the wgMLST, SNP, and core-protein genus trees, and *Pseudomonas tolaasii* was used as outgroup for 16S rRNA and core-protein family tree. A total of 28 genomes (Supplementary Table S3) was used to construct the phylogenetic family tree by the genome BLAST distance phylogeny method (GBDP) in the Type Strain Genome Server (TYGS) platform (https://tygs.dsmz.de/) [98]. All phylogenetic trees were visualized using an interactive tree of life (iTOL) v5 (https://itol.embl.de/) online tool [99].

### 4.4. Orthology and Pan-Genome Analyses

OrthoVenn2 (https://orthovenn2.bioinfotoolkits.net/home) webserver [100] was used to identify orthologous gene clusters that are unique and shared among the *E. americana* strains. The analysis was performed with default parameters for the protein-coding genes of the strains. The protein-coding genes were also clustered using USEARCH [101]. The pan-genome (core, accessory, and unique genes) of the *E. americana* strains were calculated and annotated in the COGs and KEGG databases using the Bacterial Pan Genome Analysis tool (BPGA) pipeline [102].

### 4.5. Resistome and Antimicrobial Susceptibility Profile of B6-1

The comprehensive antibiotic resistance database (CARD) (https://card.mcmaster.ca/home) [103] was used to detect antibiotic resistance genes. Antibiotic susceptibility test was done by Kirby–Bauer disk diffusion method [104] following the recommendations of the Clinical and Laboratory Standards Institute [105]. *E. americana* strain B6-1 was cultured on Mueller-Hinton Agar (MHA) (Solarbio Life sciences, Beijing, China). *Escherichia coli* ATCC 25922 was used as the control for these assays. The antibiotic discs (Oxoid, Wesel, Germany) used included ampicillin (AMP, 10 μg), ciprofloxacin (CIP, 5 μg), cefazolin (30 μg), cefixime (5 μg), erythromycin (E, 15 μg), streptomycin (S, 10 μg), kanamycin (K, 30 μg), gentamicin (10 μg), tetracycline (TET, 30 μg), rifampicin (5 μg), vancomycin (30 μg), ceftriaxone (30 μg), ofloxacin (5 μg), clindamycin (22 μg), aztreonam (30 μg) and novobiocin (5 μg). The diameters of the inhibition zone used for classifying the microorganism’s antibiotic susceptibilities are shown in Table S8. All antibiotic resistance determinations were conducted in triplicates.

### 4.6. Mobile Genetic Elements (MGE) and Bacterial Defense

Genomic islands were predicted using IslandViewer 4 [106]. Insertion sequences, plasmids, and integrons were predicted using Isfinder [107], PlasmidFinder (https://cge.cbs.dtu.dk/services/PlasmidFinder/) [108], and INTEGRALL database (http://integrall.bio.ua.pt/) [109], respectively. PHAge Search Tool—Enhanced Release (PHASTER) web server (www.phaster.ca.) [110] was used to predict bacteriophage sequences. Putative CRISPR loci and Cas clusters were examined using clustered regularly interspaced short palindromic repeats (CRISPR) (https://crisprcas.i2bc.paris-saclay.fr/CrisprCasFinder/Index) [111]. The restriction-modification system and toxin-antitoxin system (TA system) were identified from the KEGG annotation.

### 4.7. Pathogenicity, and Virulence Factors

BLASTP (E-value cutoff of 1 × 10^−5^) was used to detect virulence genes detected by searching against the virulence factor database (VFDB; http://www.mgc.ac.cn/VFs/) [112]. Pathogenfinder (https://cge.cbs.dtu.dk/services/PathogenFinder/) was used to predict pathogenicity towards humans [113]. Type secretion systems were detected using MacSyFinder v1.0.2 [114]. Genes related to dormancy, sporulation, and stress response were inferred from the KEGG annotation.

### 4.8. CAZymes and Secondary Metabolites

Carbohydrate-active enzymes (CAZymes) and secondary metabolites gene clusters were predicted using the dbCAN2 meta server (http://cys.bios.niu.edu/dbCAN2) [115] and antiSMASH 5.0 (https://antismash.secondarymetabolites.org/#!/start) [116], respectively. BAGEL4 (http://bagel4.molgenrug.nl/) web server [117] was used to annotate bacteriocins found in the genomes of the five *E. americana* strains. 

## 5. Conclusions

In this study, we performed a comparative genomic analysis of five *E. americana*. There were significant differences in the genome size and number of predicted genes between strains isolated from different host-habitats. Our phylogenetic analysis revealed that the strains formed clusters according to their host and evolution, but the habitat was involved in shaping the genomes of the strains. The pan-genome analysis revealed conserved and variable genes involved in all fundamental life processes, including growth, development, virulence, antibiotic resistance, detoxification, and adaptation to host and environment. Additionally, all the *E. americana* strains are weak pathogens and/but contain genes from virulence factors, macromolecular secretion system, toxins, antibiotic resistance, CAZymes, secondary metabolites, and stress response that aid the pathogen to colonize hosts across kingdoms and different environments. 

Further analysis revealed that the acquisition of mobile genetic elements is a significant source of genome diversity in the genus and possesses highly conserved defense systems made up of CRISPR elements, R-M system, and TA systems. The rhamnose biosynthesis, R-M system, and TA system are potential targets for future new drugs to control the bacterium. Additionally, the *E. americana* strains have putative genes for the production of bacteriocin and the biodegradation of toxic compounds. This result provides the opportunity for the development and commercialization of useful products such as control of multidrug-resistant bacteria and bioremediation. However, further work is required to ascertain the production of these compounds and testing to validate their efficacy. 

The findings suggest that multiple high-quality genome sequences of the pathogen from a different host and geographical location are required to understand the virulence and genetic factors that allow the *Ewingella* group to be versatile and adapt to a broad niche. This work suggests the optimization of commercial mushroom production processes to minimize the use of antibiotics, including whole-genome sequencing techniques, as routine testing for mushroom quality and safety to minimize the potential risk to human health.

## Figures and Tables

**Table 1:** Antimicrobial susceptibility profiles of strain B6-1.

Antibiotic Class.	Antimicrobial Agent	* Susceptibility (Average Diameter/mm)
β-Lactams	Ampicillin	R (11.10)
Cephalosporins	Aztreonam	S (23.34)
	Cefazolin	R (11.78)
	Cefixime	I (17.69)
	Ceftriaxone	S (21.51)
Fluoroquinolone	Ciprofloxacin	S (23.75)
	Ofloxacin	S (23.33)
Aminoglycosides	Streptomycin	S (14.87)
	Gentamicin	S (17.85)
	Kanamycin	S (19.67)
Large ring lactone	Erythromycin	I (14.05)
Tetracycline	Tetracycline	R (12.64)
Rifamycin	Rifampicin	R (8.54)
Lincosamide	Clindamycin	R (0.00)
Sugar peptide	Vancomycin	R (0.00)
	Novobiocin	R (0.00)

* R, Resistance to the antibiotics; S, Susceptible to the antibiotics; I, intermediate resistance to the antibiotics. The values in bracket represent the diameter in mm of bacteria growth, which was used to classify the susceptibility to antibiotics. * The classification of each antibiotic susceptibility levels can be found in Supplementary Table S8.

**Table 2:** Total number of Integron, plasmids, genome islands CRISPR, R-M system and TA system in the *E. americana* strains.

Strain	Integron (In1)	Insertion Sequence	Plasmid	GI	CRISPR Spacers	R-M System	TA System		
							Type I	Type II	Type IV
B6-1	4	2	2	19	4	I, II, IV	2	22	3
ATCC	4	4	0	20	5	I, II	0	29	1
CCUG	4	4	0	23	4	I, II	0	26	1
NCTC	4	4	0	24	4	I, II	0	30	1
RIT713	5	2	0	23	2	I, II, IV	1	30	4

**Table 3:** The distribution of phages among the *E. americana* strains.

Strain Name	Region	Length (kb)	Completeness	No. of CDS	GC%	Predominant Phage
B6-1	1	43.9	Intact	52	51.81	Entero_mEp390_NC_019721
	2	17.9	Intact	25	52.35	Erwini_ENT90_NC_019932
ATCC	3	51.8	Intact	48	50.54	Entero_mEp460_NC_019716
CCUG	1	35.8	Intact	43	51.88	Haemop_HP1_NC_001697
	1	44.5	Intact	51	50.42	Entero_mEp460_NC_019716
NCTC	1	24.8	Questionable	37	50.50	Entero_mEp460_NC_019716
	2	33.6	Intact	24	53.04	Entero_N15_NC_001901
	1	35.6	Intact	45	51.93	Aeromo_phiO18P_NC_009542
	2	18.1	Intact	24	54.42	Erwini_ENT90_NC_019932
RIT713	1	47.0	Intact	83	49.9	Edward_GF_2_NC_026611
	1	35.6	Intact	45	51.93	Aeromo_phiO18P_NC_009542
	2	18.1	Intact	24	54.42	Erwini_ENT90_NC_019932

**Table 4:** The predicted pathogenicity score for the *Ewingella americana* strains from the PathogenFinder.

Strain	Host	Predicted Pathogenicity Score	Probability of Being a Human Pathogen	Pathogenic Families Matched	Being a Human Pathogen
B6-1	*F*. *filiformis*	46.24	0.60	24	Yes
RIT713	*Craterellus* sp.	53.60	0.64	24	Yes
ATCC	Human	46.21	0.61	24	Yes
CCUG	Human	48.41	0.62	24	Yes
NCTC	Human	54.71	0.63	25	Yes
*Yersinia pestis* CO92	Human	6174.52	0.91	1569	Yes

**Table 5:** Total CAZymes found in the *E. americana* genomes.

CAZYme	*E. americana* Strains				
Module	B6-1	ATCC	CCUG	NCTC	RIT713
AA	6	6	6	6	7
CBM	13	13	13	12	14
CE	16	15	16	15	15
GH	70	68	68	73	65
GT	53	52	52	60	48
PL	2	2	2	2	2
**Total**	**160**	**156**	**157**	**168**	**151**

## Supplementary Materials

The following are available online at https://www.mdpi.com/2076-0817/9/5/330/s1, Figure S1: The quality of genome from Quast, Figure S2: The pan-genome analysis of five *Ewingella americana* strains, Figure S3: Phylogenetic analysis of the five strains of *Ewingella americana* and other closely related neighbors in the family Yer-siniaceae, Table S1: Physiological and biochemical characteristics of Bacterium causing brown rot disease of *Flammulina filiformis*, Table S2: List of Bacterial species used for 16S rRNA gene phylogenetic tree, Table S3: The List of Bacterial Species Used in Phylogenetic family tree, Table S4: The ANI and dDDH values for the *Ewingella americana* strains and other closely related species, Table S5: The Tetra Correlation Search (TCS) calculations for E4, Table S6: The number core, accessory, and unique genes in the *Ewingella americana* pan-genome, Table S7: The number of putative antibiotic resistant genes for the *Ewingella americana* strains identified from search against the CARD database, Table S8: Evaluation standards for antibiotic sensitivity, Table S9: The number of Class I integron found in the genomes of the *Ewingella americana* strains, Table S10: The type of insertion sequences in the genomes of the *Ewingella americana* strains, Table S11: SwissProt annotation of plasmids in *Ewingella americana* B6-1, Table S12: The number of toxin-antitoxin system-related genes found in the genomes of *Ewingella americana* strains; Table S13: The number of putative virulence factor genes for the *Ewingella americana* strains identified from search against the VFDB database, Table S14: The number of macromolecular secretion system genes for the *Ewingella americana* strains, Table S15: The number of stress response related genes found in the genomes of *Ewingella americana* strains, Table S16: List of secondary metabolites found in the genomes of *Ewingella americana* strains using antiSMASH.

## Abbreviations

B6-1	*Ewingella americana* B6-1
ATCC	*Ewingella americana* ATCC 33852
CCUG	*Ewingella americana* CCUG 14506T
NCTC	*Ewingella americana* NCTC12157
RIT713	*Ewingella americana* RIT713
E4	E4
*E. americana*	*Ewingella americana*
ANI	Average Nucleotide Identity
CAZyme	Carbohydrate-active enzymes
CRISPR	Clustered regularly interspaced short palindromic repeats
dDDH	digital DNA-DNA hybridization
GC	Guanine-cytosine
MGE	Mobile genetic elements
NCBI	National Center for Biotechnology Information
SMs	Secondary metabolites
SNP	Single-nucleotide polymorphism
R-M system	Restriction-modification system
TA system	Toxin-antitoxin system
