# Supplementary material for: Comparative Genomic Analysis Provides Insights into the Phylogeny, Resistome, Virulome, and Host Adaptation in the Genus Ewingella
Source: Pathogens. 2020 Apr 28;9(5):330. doi: 10.3390/pathogens9050330 (PMC7281767; doi:10.3390/pathogens9050330)
Supplement: Supplementary file 1 [file pathogens-09-00330-s001.zip › FigureS3.pdf]

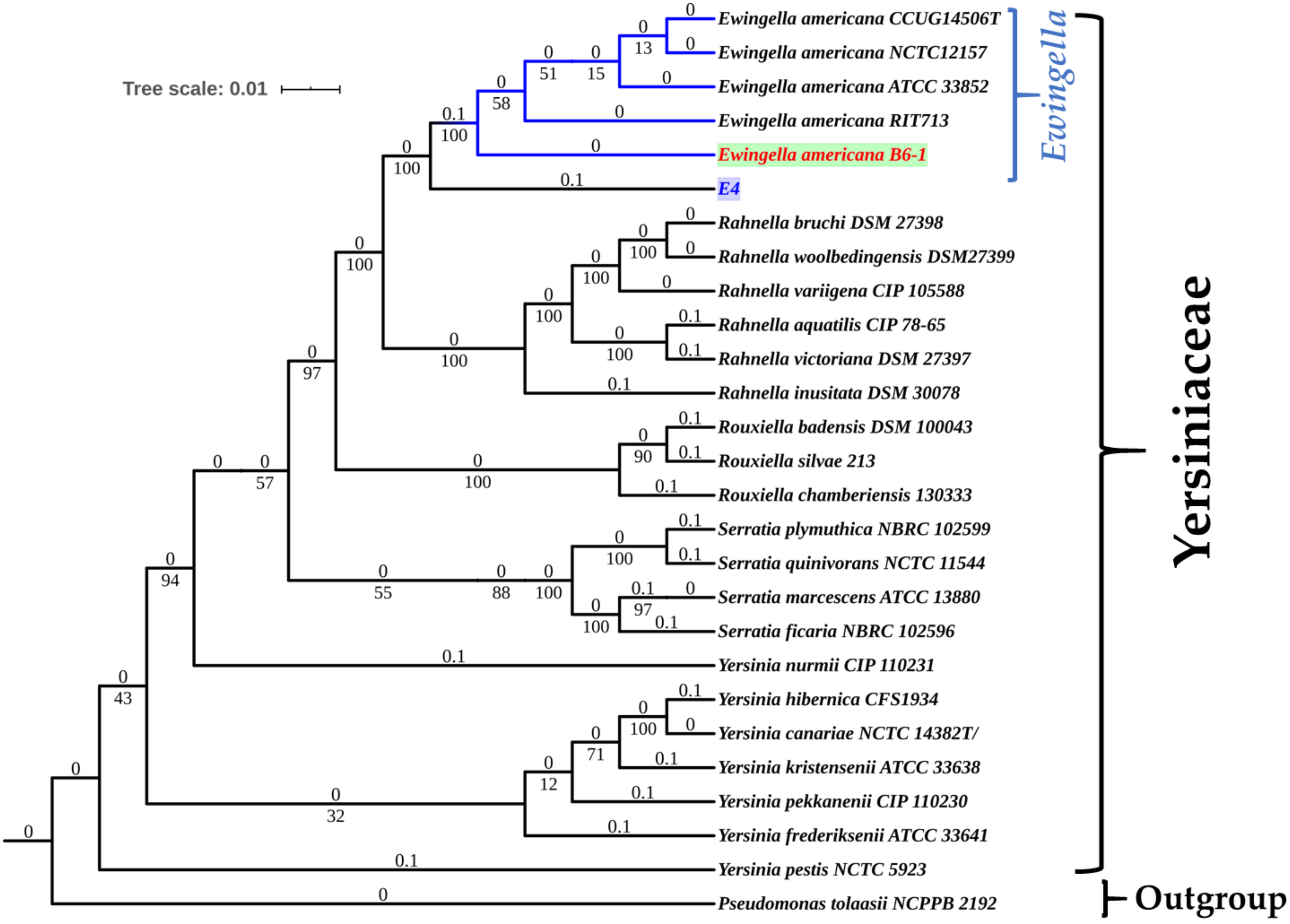

Figure S3. Phylogenetic analysis of the five strains of *Ewingella americana* and other closely related neighbors in the family Yersiniaceae. *Pseudomonas tolaasii* was used as an outgroup. Scale bar indicates the average number of nucleotides per substitution.
