# Supplementary material for: Comparative Genomic Analysis Provides Insights into the Phylogeny, Resistome, Virulome, and Host Adaptation in the Genus Ewingella
Source: Pathogens. 2020 Apr 28;9(5):330. doi: 10.3390/pathogens9050330 (PMC7281767; doi:10.3390/pathogens9050330)
Supplement: Supplementary file 1 [file pathogens-09-00330-s001.zip › Figure S2.pdf]

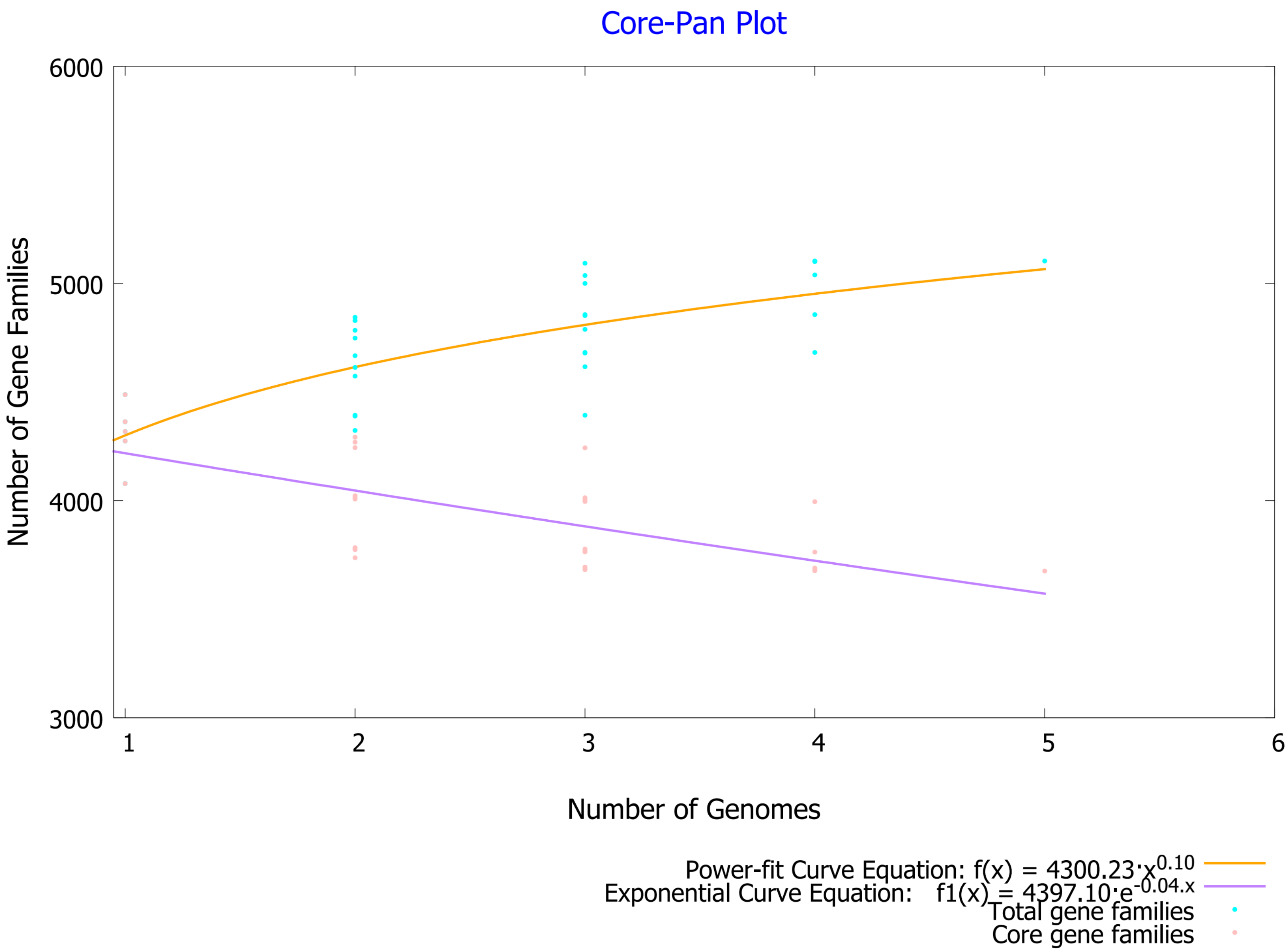

Figure S2. The pan-genome analysis of five *Ewingella americana* strains. The number of gene clusters in the pan-genome (orange line) and core-genome (purple line) plotted against the number of *E. americana* genomes
