# Supplementary figures and images for: Comparative Genomic Analysis Provides Insights into the Phylogeny, Resistome, Virulome, and Host Adaptation in the Genus Ewingella
Source: Pathogens. 2020 Apr 28;9(5):330. doi: 10.3390/pathogens9050330 (PMC7281767; doi:10.3390/pathogens9050330)

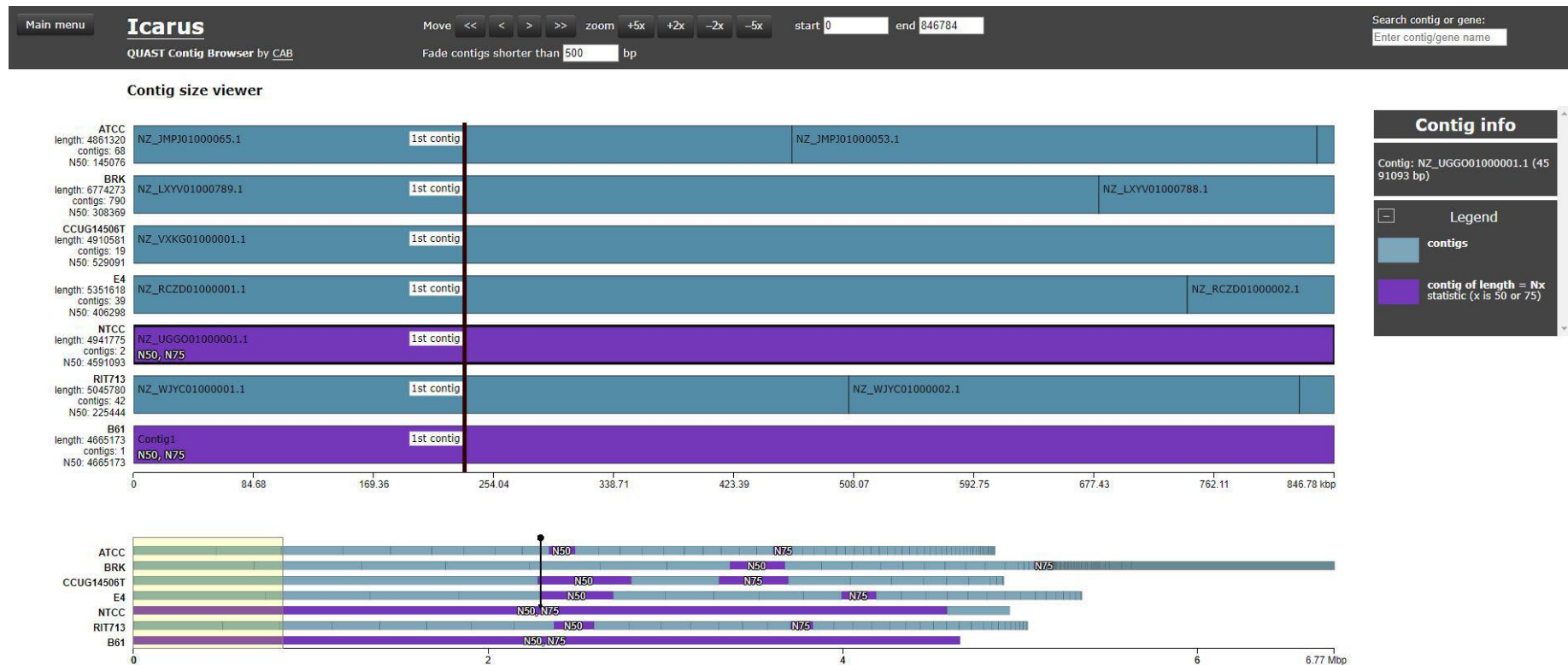

**Figure S1** The quality of the genomes of *Ewingella* sp.

Supplement: Supplementary file 1 [file pathogens-09-00330-s001.zip › Figure S1.pdf]
